# Biodistribution and Efficacy of Human Adipose-Derived Mesenchymal Stem Cells Following Intranodal Administration in Experimental Colitis

**DOI:** 10.3389/fimmu.2017.00638

**Published:** 2017-06-08

**Authors:** Mercedes Lopez-Santalla, Pablo Mancheño-Corvo, Amelia Escolano, Ramon Menta, Olga DelaRosa, Jose Luis Abad, Dirk Büscher, Juan M. Redondo, Juan A. Bueren, Wilfried Dalemans, Eleuterio Lombardo, Marina I. Garin

**Affiliations:** ^1^Division of Hematopoietic Innovative Therapies, Centro de Investigaciones Energéticas, Medioambientales y Tecnológicas (CIEMAT), Centro de Investigación Biomédica en Red de Enfermedades Raras (CIBER-ER), Madrid, Spain; ^2^Advanced Therapies Mixed Unit, Instituto de Investigación Sanitaria Fundación Jiménez Díaz (IIS-FJD), Madrid, Spain; ^3^TiGenix SAU, Madrid, Spain; ^4^Gene Regulation in Cardiovascular Remodeling and Inflammation Laboratory, Centro Nacional de Investigaciones Cardiovasculares Carlos III (CNIC), Madrid, Spain; ^5^Coretherapix, Madrid, Spain; ^6^Grifols, Barcelona, Spain; ^7^TiGenix NV, Leuven, Belgium

**Keywords:** adipose-derived mesenchymal stem cells, intranodal therapy, colitis, biodistribution, efficacy, immunomodulation

## Abstract

Mesenchymal stem cells (MSCs) have a large potential in cell therapy for treatment of inflammatory and autoimmune diseases, thanks to their immunomodulatory properties. The encouraging results in animal models have initiated the translation of MSC therapy to clinical trials. In cell therapy protocols with MSCs, administered intravenously, several studies have shown that a small proportion of infused MSCs can traffic to the draining lymph nodes (LNs). This is accompanied with an increase of different types of regulatory immune cells in the LNs, suggesting the importance of migration of MSCs to the LNs in order to contribute to immunomodulatory response. Intranodal (IN), also referred as intralymphatic, injection of cells, like dendritic cells, is being proposed in the clinic for the treatment of cancer and allergy, showing that this route of administration is clinically safe and efficient. In this study, we investigated, for the first time, the biodistribution and the efficacy of Luciferase^+^ adipose-derived MSCs (Luci-eASCs), infused through the inguinal LNs (iLNs), in normal mice and in inflamed mice with colitis. Most of the Luci-eASCs remain in the iLNs and in the adipose tissue surrounding the inguinal LNs. A small proportion of Luci-eASCs can migrate to other locations within the lymphatic system and to other tissues and organs, having a preferential migration toward the intestine in colitic mice. Our results show that the infused Luci-eASCs protected 58% of the mice against induced colitis. Importantly, a correlation between the response to eASC treatment and a higher accumulation of eASCs in popliteal, parathymic, parathyroid, and mesenteric LNs were found. Altogether, these results suggest that IN administration of eASCs is feasible and may represent an effective strategy for cell therapy protocols with human adipose-derived MSCs in the clinic for the treatment of immune-mediated disorders.

## Introduction

Mesenchymal stem cells (MSCs) are multipotent adult stem cells capable of self-renewal and differentiation into multiple cell lineages of mesodermal origin such as chondrocytes, adipocytes, and osteocytes (1). These cells have been identified in multiple tissues such as bone marrow (2), umbilical cord blood (3), amniotic fluid (4), adipose tissue (5), dental pulp (6), and among others. MSCs are considered as a novel modality of therapy for a wide variety of degenerating and immunological disorders (1, 2, 7, 8). The encouraging results in animal models have initiated the translation of MSC therapy in clinical trials in a range of immunomediated diseases such as graft versus host disease (9), inflammatory bowel disease (10–12), multiple sclerosis (13), systemic lupus erythematosus (14, 15), rheumatoid arthritis (16–20), and among others. The immunomodulatory capacity of MSCs takes place both by direct cell-to-cell contact and by means of soluble factors (21). At present, it is unknown whether the therapeutic effects of MSCs are carried out by a small fraction of the administered MSCs that migrates to sites of inflammation or/and through distant induction of regulatory immune cells that mediate immunomodulation and tissue repair (22). There are controversial data regarding the presence of MSCs at site of inflammation (23–25). In any case, their therapeutic effects are linked to a final increase of myeloid and lymphoid cells with a regulatory phenotype in the secondary lymphoid tissues associated to the inflammation site (18, 19, 25, 26). Thus, in this study (27), we hypothesized that intranodal (IN) administration of MSCs could be feasible and would represent an alternative route for modulating immune responses (18, 25, 28). IN administration treatments are being extensively used in clinical trials for the treatment of cancer (29–32) and allergy (33, 34), indicating that this route of administration is feasible in humans and can be applied to the clinic. Data from clinical trials show that IN therapy is easy, practically painless, safe and is associated with a low risk of systemic adverse effects (35–37). Additionally, Gil-Ortega et al. provided robust evidences that endogenous adipose-derived MSCs (eASCs) can navigate through the lymphatic system (28). At present, IN administration of MSCs has not been assessed previously, whereas intravenous administration of MSCs has been used in many experimental models and in clinical trials. However, the intravenous injection of MSCs is thought to be limited by cell entrapment in the lungs (24, 38). This might limit the bioavailability of the cells, their trafficking to tissues and, possibly, their therapeutic effects (24). Local administration of MSCs is of interest in situations where they can be directly implanted in the injured tissues trying to improve their beneficial effects (39–42). The number of MSCs that can be delivered inside the lymph node (LN) after IN administration could be significantly greater than the number of cells that reach the LNs following their delivery through the blood stream, either through intravenous or intraperitoneal administration. This may allow achieving robust therapeutic effects while potentially reducing the cell dose of MSCs required for modulating inflammation.

In this study, we aimed to examine the biodistribution of human Luciferase^+^ eASCs (Luci-eASCs) after IN administration in steady state and following an acute inflammatory challenge in the colon with the haptenizing reagent trinitrobenzene sulfonic acid (TNBS). Strikingly, modulation of intestinal inflammation was observed. In parallel to this study, we also demonstrate that IN infusion of eASCs can modulate ongoing acute inflammatory responses in an experimental model of collagen-induced arthritis (27).

## Materials and Methods

### Mice

C57/BL6 male mice of 6–8 weeks of age were obtained from Charles River (Burlington, MA, USA). All experiments were performed in accordance with the corresponding regulations regarding experimental animal welfare (RD 223/1998 and Directive 2010/63/EU protocols).

### Generation of Human Expanded Adipose-Derived MSCs

Human samples were obtained after informed consent as approved by the Spanish Ethics Committee of reference for the site of tissue procurement (Clínica de la Luz Hospital, Madrid, Spain). Human adipose tissue aspirates from healthy donors were processed as described elsewhere (18). All the eASCs used fulfilled the release criteria of identity, purity, and potency needed for their clinical use.

### Generation of Luci-eASCs

Reporter Luciferase-EGFP bicistronic retroviral vector was constructed using standard cloning procedures. The H2B gene was amplified by PCR (forward, TATGGGTACCGCCACCATGCCAGAGCCAGCGAAG; reverse, TATGGATCCTTAGCGCTGGTGTACTTG) and cloned into pCopGFP2i-N (Evrogen, Moscow, Russia) using *Acc*65I–*Bam*HI sites. Internal ribosome entry site (IRES) element was isolated from pIRES2-EGFP (Clontech, Mountain View, CA, USA) using *Sal*I–*Msc*I and cloned upstream H2B-CopGFP2i into *Sal*I–*Acc*65I (Klenow blunted) sites. Luciferase was cut from pGL2 Basic (Promega, Madison, WI, USA), using *Xho*I–*Xba*I (Klenow blunted) restriction sites, and inserted upstream IRES-H2B-CopGFP2i into *Xho*I–*Sal*I (Klenow blunted). CopGFP2i was then replaced by EGFP, from pEGFP-N1 (Clontech) using *Age*I–*Not*I sites. The Luciferase-IRES-H2B-EGFP cassette was separated using *Bgl*II–*Not*I and cloned into *Bam*HI–*Not*I sites of pRV retroviral vector to give the final pRV-Luciferase-IRES-H2B-EGFP bicistronic reporter construction. Transfection and generation of viral supernatants were performed using polyethylenimine (1 vol PEI:2 vol DNA). Viral supernatants were used for infection of eASCs. For infection, eASCs were seeded in 6-well plates and infected with retroviral particles concentrated when cell confluency was between 70 and 80%. Finally, the transduction was evaluated by flow cytometry (FACS Calibur™, BD Bioscience, San Diego, CA, USA), selecting clones with 90% expression of EGFP by cell sorting.

### Immunophenotyping of Luci-eASCs

Luci-eASCs were defined according to the criteria of the International Society for Cellular Therapy (43). Luci-eASCs were characterized by their immunophenotype; positive for CD73 (AD2), CD90 (5E10, both from Becton Dickinson), and CD105 (43A3, from Biolegend) and negative for CD14 (RM052, from Immunotech), CD34 (8G12, from Becton Dickinson), CD45 (J33, from Beckman Coulter), and HLA-DR (AF6-120.1, from eBiosciences).

### Immunosuppression Assay of Luci-eASCs

Buffy coats were provided by the National Transfusion Centre of the Comunidad Autonoma of Madrid. Peripheral blood mononuclear cells (PBMCs) were isolated by density centrifugation gradient using Ficoll-Paque Plus (GE Healthcare Biosciences AB, Uppsala, Sweden) following the supplier’s protocol. Purity was verified by flow cytometry.

For CFSE labeling, PBMCs were washed extensively to remove fetal bovine serum (FBS), resuspended in a 10 µM CFSE (Carboxyfluorescein diacetate *N*-succinimidyl ester, Sigma-Aldrich, St. Louis, MO, USA) solution (10^7^ PBMC per 200 µL of solution), and incubated under constant shaking at 37°C for 10 min. Reaction was stopped by adding ice-cold medium (RPMI + 10% FBS), and cells were washed three times with ice-cold phosphate buffer saline. Cells were then cultured overnight and one aliquot was used to set up and control the FL-1 voltage for CFSE. After resting overnight, CFSE-labeled PBMCs were activated with the Pan T Cell Activation Kit (microbeads coated with anti-CD3, anti-CD2, and anti-CD28; Miltenyi Biotech, Auburn, CA, USA) following the manufacturer’s instructions. PBMCs (10^6^ cells/well) were cultured in 24-well plates alone or with Luci-eASCs (4 × 10^4^ cells/well; Luci-eASC:PBMC ratio 1:25) in a total volume of 1 mL of RPMI + 10% FBS. The 1:25 ratio was chosen because it provided a high inhibitory effect, on the basis of previous studies (44, 45). After 5 days, PBMCs were harvested and labeled with 7-AAD and anti-CD3 antibody, and cell proliferation of the CD3^+^/7-AAD^−^ population (viable CD3 T lymphocytes) was determined by flow cytometry, according to loss of CFSE signal. Data were analyzed with the use of FCSExpress 4 (*De Novo* Software, Glendale, CA, USA) and BD CellQuest™ Pro analysis (Becton Dickinson) softwares. CaliBRITE beads (BD Bioscience, Erembodegem-Aalst, Belgium) were used before each assay to calibrate the cytometer.

### Induction and Evaluation of Colitis after Treatment with Luci-eASCs

To induce colitis, male C57BL/6 mice (6–8 weeks old) were preimmunized on their shaved back with 1% of TNBS (Sigma-Aldrich, St. Louis, MO, USA) in a mixture of acetone:olive (4:1 v/v). After 1 week, colitis was induced by intrarectal administration of 3 mg of TNBS in 50% ethanol (100 µL) per mouse. 1 h after TNBS administration, 3.2 × 10^5^ of Luci-eASCs per mouse were administered IN, by injection of the cell suspension in the iLNs (1.6 × 10^5^ cells in both the right and the left iLNs). As controls, healthy mice infused with the Luci-eASC and mice treated with intrarectal 50% EtOH only (vehicle of the TNBS) and infused with Luci-eASC IN were used. Score of colitis (weight of mice, stools, and general aspect of mice) was monitored for 48 h.

### *In Vivo* Optical Bioluminescence Imaging

Bioluminescent imaging analysis was conducted at 48 h after the infusion of Luci-eASCs with the IVIS 200 imaging system (Caliper, Hopkinton, MA, USA).

Whole-body bioimaging analysis was done in anesthetized mice. The bioimaging analysis in main tissues and organs (liver, spleen, intestine, lungs, heart, and blood), secondary LNs (popliteal, popLN; parathymic, thymLN; parathyroid, thyrLN; mesenteric, mLN; caudal, cLN; axillary, axLN; and inguinal, iLN, LNs) and the adipose tissue around the inguinal LNs, where the Luci-eASCs were injected, were determined immediately after culling the mice. Photons emitted were acquired as photons per s/cm^2^ per steradian using Living Imaging 3.0 (Caliper). For photon quantification, a region of interest was manually selected and kept constant within each experiment.

Bioluminescence signal was analyzed as percentage of light units per tissue relative to the total number of light units per mouse. Total bioluminescence signal for LNs, tissues, and organs were calculated as the sum of the light units in each tissue. Values of bioluminescence signal below 10,000 light units were considered negative.

### Statistical Analysis

Data are presented as the interquartile range (p75, upper edge; p25, lower edge; p50, midline; p95, line above the box, and p5, line below the box). Normal distribution was analyzed by the Shapiro-Wilks test. Non-parametric techniques (Mann–Whitney *U* test) were used (with Bonferroni adjustment). Analysis was performed using the software Stata 11 (StataCorp, USA) and GraphPad Prism 5.00 for Windows program (GraphPad Software, San Diego, CA, USA).

## Results

### The Majority of the Intranodally Injected Luci-eASCs Remain in the LNs 48 h Postinfusion

In the present study, we investigated the biodistribution of the Luci-eASCs after IN administration in an experimental model of acute intestinal inflammation induced by TNBS which displays human inflammatory bowel disease-like clinical, histopathologic, and immunologic features (46).

Human adipose-derived MSCs expressing the luciferase gene were generated by using retrovirus-based methods (See Materials and Methods). To confirm the ASC characteristics of the transduced Luci-eASCs, immunophenotyping by flow cytometry and *in vitro* immunosuppression assays were carried out. As expected, Luci-eASCs were positive for CD73, CD90, and CD105 and negative for CD14, CD34, CD45, and HLA-DR according to the criteria of the International Society for Cellular Therapy (43) (Figure S1A in Supplementary Material). To evaluate the immunosuppression capacity of Luci-eASCs, T-cell proliferation assays were carried out with CFSE-labeled PBMCs stimulated with microbeads coated with anti-CD3/CD2/CD28 antibodies in the presence or absence of untransduced eASCs or Luci-eASCs (ratio eASCs:PBMCs, 1:25). Proliferation of viable CD3^+^ T cells was determined at 120 h by flow cytometry. Luci-eASCs inhibited T-cell proliferation at similar levels as the untransduced eASCs (Figure S1B in Supplementary Material).

Initial experiments were set up to define for how long the bioluminescence signal of the infused Luci-eASCs can be detected *in vivo*. A total of 3.2 × 10^5^ Luci-eASCs were injected through the inguinal LNs into healthy mice. The bioluminescence signals were monitored at different time points. As shown in Figure S2A in Supplementary Material, the bioluminescence signal declined very rapidly within the first 72 h period from the infusion of the Luci-eASCs. Therefore, 48 h postinfusion of Luci-eASCs was chosen as the best time-point that allows analyzing *in vivo* the biodistribution of the infused injected Luci-eASCs.

**Figure 1:**
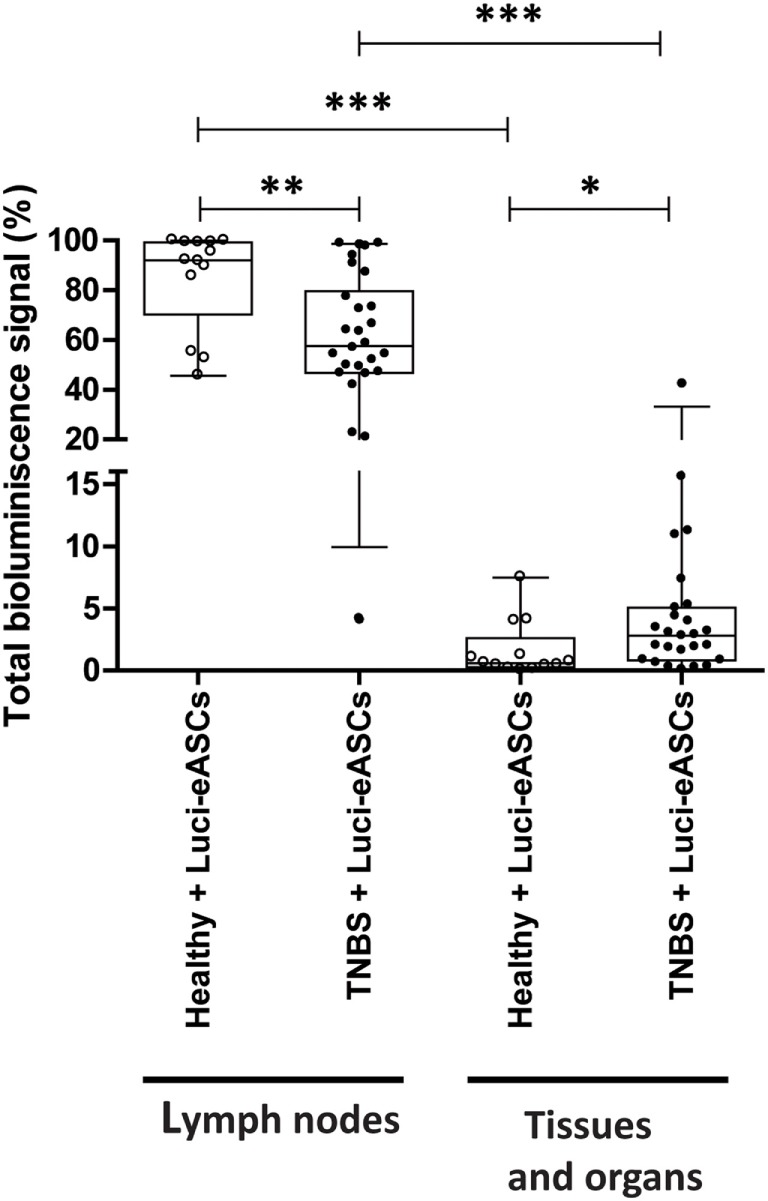
Analysis of total bioluminescence signal of Luci-eASCs in lymph nodes (LNs) and in tissues and organs at 48 h following intranodal administration. Sums of bioluminescence signals measured at 48 h as percentage of light units per LN (inguinal, popliteal, parathymic, parathyroid, mesenteric, caudal, and axillary included) and per tissue and organ (liver, spleen, intestine, lungs, heart, and blood included) relative to the total number of light units per mouse were expressed. Data are presented by dots and box-plots that represent the interquartile range (p75, upper edge; p25, lower edge; p50, midline; p95, line above the box; and p5, line below the box) of the percentage of total bioluminescence signal. Significance was analyzed by the Mann–Whitney *U* test and represented by *p < 0.05, **p < 0.01, and ***p < 0.001. Results correspond to four independent experiments.

At 48 h postinfusion of the Luci-eASCs, the highest bioluminescence signals were measured in the LNs analyzed in this study (popLN, thymLN, thyrLN, mLN, cLN, axLN, and iLN), both in healthy [92.0 (70.3–99.2)%] and in TNBS-colitic mice [57.5 (46.8–79.6)%], whereas lower levels of the bioluminescence signals were detected in other organs and tissues analyzed (liver, spleen, intestine, lungs, heart, and blood) in both healthy [0.6 (0.3–2.6)%] and TNBS-colitic mice [2.8 (0.8–5)%] (Figure 1; Figure S2 in Supplementary Material). Interestingly, in TNBS-colitic mice, a significant increase of the bioluminescence signal in the tissues and organs studied was detected in comparison with healthy mice. This paralleled with a decrease in the bioluminescence signal in the LNs of TNBS-colitic mice when compared to healthy mice (Figure 1). These results show that the majority of the infused Luci-eASCs remain in the LNs, 48 h postinfusion and that the inflammatory challenge with TNBS, enhance the trafficking of a small, but significant, proportion of the infused-Luci-eASCs to other tissues and organs.

### Intranodal Administered Luci-eASCs Remained in the iLNs and, Upon Inflammation, a Small Proportion of Luci-eASCs Traffics to the Intestine

**Figure 2:**
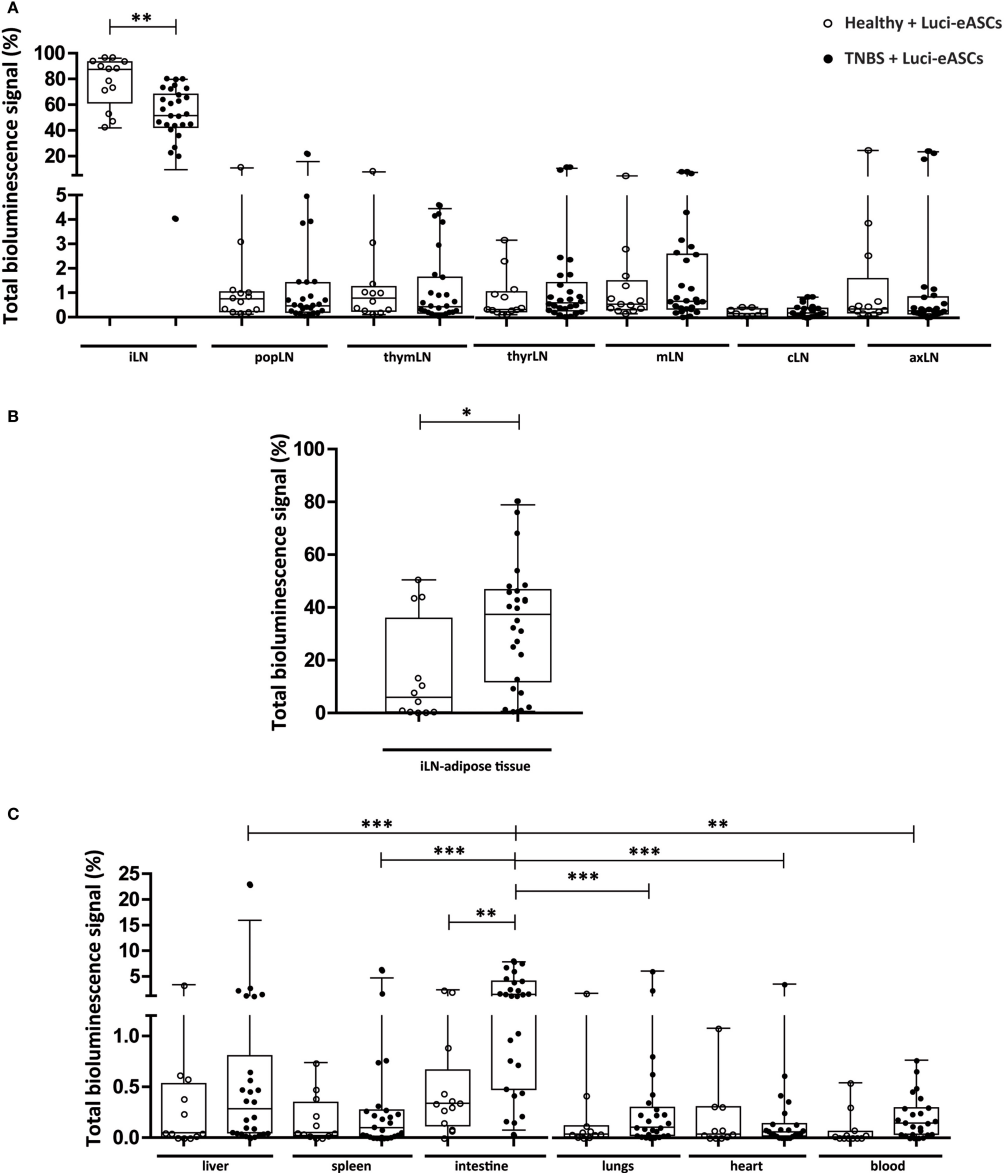
Analysis of total bioluminescence signal of Luci-eASCs in the different lymph nodes (LNs) (inguinal, popliteal, parathymic, parathyroid, mesenteric, caudal, and axillary), adipose tissue adjacent the inguinal LNs and main tissues and organs (liver, spleen, intestine, lungs, heart, and blood) at 48 h. Bioluminescence signals measured at 48 h in the inguinal LNs (iLN), popliteal (popLN), parathymic (thymLN), parathyroid (thyrLN), mesenteric (mLN), caudal (cLN), and axillary (axLN) **(A)**, adipose tissue surrounding the iLNs **(B)**, and in the liver, spleen, intestine, lungs, heart, and blood **(C)** [Healthy + Luci-eASCs intranodal (IN), *n* = 12; TNBS + Luci-eASCs IN *n* = 25] were expressed as percentage of light units per tissue relative to the total number of light units per mouse. Data are presented by dots and box-plots that represent the interquartile range (p75, upper edge; p25, lower edge; p50, midline; p95, line above the box, and p5, line below the box) of the percentage of total bioluminescence signal. Significance was analyzed by the Mann–Whitney *U* test and represented by *p < 0.05, **p < 0.01, and ***p < 0.001. Results correspond to four independent experiments.

To further dissect the biodistribution of the Luci-ASCs and how an acute inflammatory challenge may impact their homing toward the inflamed tissues, the bioluminescence signals were analyzed separately in the LNs (inguinal, popliteal, thymic, thyroid, mesenteric, caudal, and axillary LNs) and in the tissues and organs (liver, spleen, intestine, lung, heart, and peripheral blood). As shown in Figure 2A and Figure S2C in Supplementary Material, when Luci-eASCs were administered IN, most of the bioluminescence signal was found in the iLNs [87.5 (61.5–93.2)% in healthy mice and 51.4 (42.3–68.1)% in TNBS-colitic mice, *p* = 0.0011] where the Luci-eASCs were injected. In contrast, very low levels of the bioluminescence signals [0.4 (0.2–1.3)%] were measured within the lymphatic system with no clear preference for any of the LNs analyzed (Figure 2A).

Moreover, high bioluminescence signals were also detected in the adipose tissue surrounding the iLNs [5.9 (0.3–35.9)% in healthy mice and 37.4 (11.9–46.7)% in TNBS-colitic mice, Figure 2B; Figure S2D in Supplementary Material]. This suggests that after IN administration of the Luci-eASCs, the majority of the cells remained at the injection site either inside the LNs or in the adipose tissue associated to the LNs.

The bioluminescence signals in the other tissues and organs analyzed were very low (below 3%). The bioluminescence signal found in the intestine was significantly increased in TNBS-colitic mice [1.5 (0.5–4)%] in comparison with the healthy mice [0.3 (0.1–0.7)%, *p* = 0.0037]. Increased bioluminescence signals, but not significant, were found in the liver [0.28 (0.04–0.80)%], spleen [0.10 (0.01–0.27)%], lungs [0.10 (0.02–0.30)%], and blood [0.14 (0.03–0.30)%] of the TNBS-colitic mice in comparison with the healthy mice [0.050 (0.008–0.531)%, 0.05 (0.02–0.35)%, 0.04 (0.01–0.12)%, and 0.009 (0.000–0.065), respectively, Figure 2C; Figure S2D in Supplementary Material]. Additionally, the bioluminescence signal found in the intestine [1.5 (0.5–4)%] was significantly increased in comparison with the other tissues and organs analyzed in the TNBS-colitic mice [0.28 (0.04–0.80)% for liver, 0.10 (0.01–0.27)% for spleen, 0.10 (0.02–0.30)% for lungs, 0.038 (0.001–0.305)% for heart, and 0.14 (0.03–0.30)% for blood].

Overall, these data indicate that when the Luci-eASCs were administered IN, the majority of the cells remained at the site of injection, either in the iLNs or the adipose tissue adjacent the iLNs. Only 0.4 (0.2–1.3)% of the bioluminescence signal was found in the rest of LNs analyzed with no clear preference for any of them. Although the amount of Luci-eASCs that migrate to the other tissues and organs analyzed was very low, there was a significant increase in the bioluminescence signal in the intestine in TNBS-colitic mice.

### Expanded Adipose-Derived MSC Administration by the IN Route Protects against TNBS-Induced Colitis

**Figure 3:**
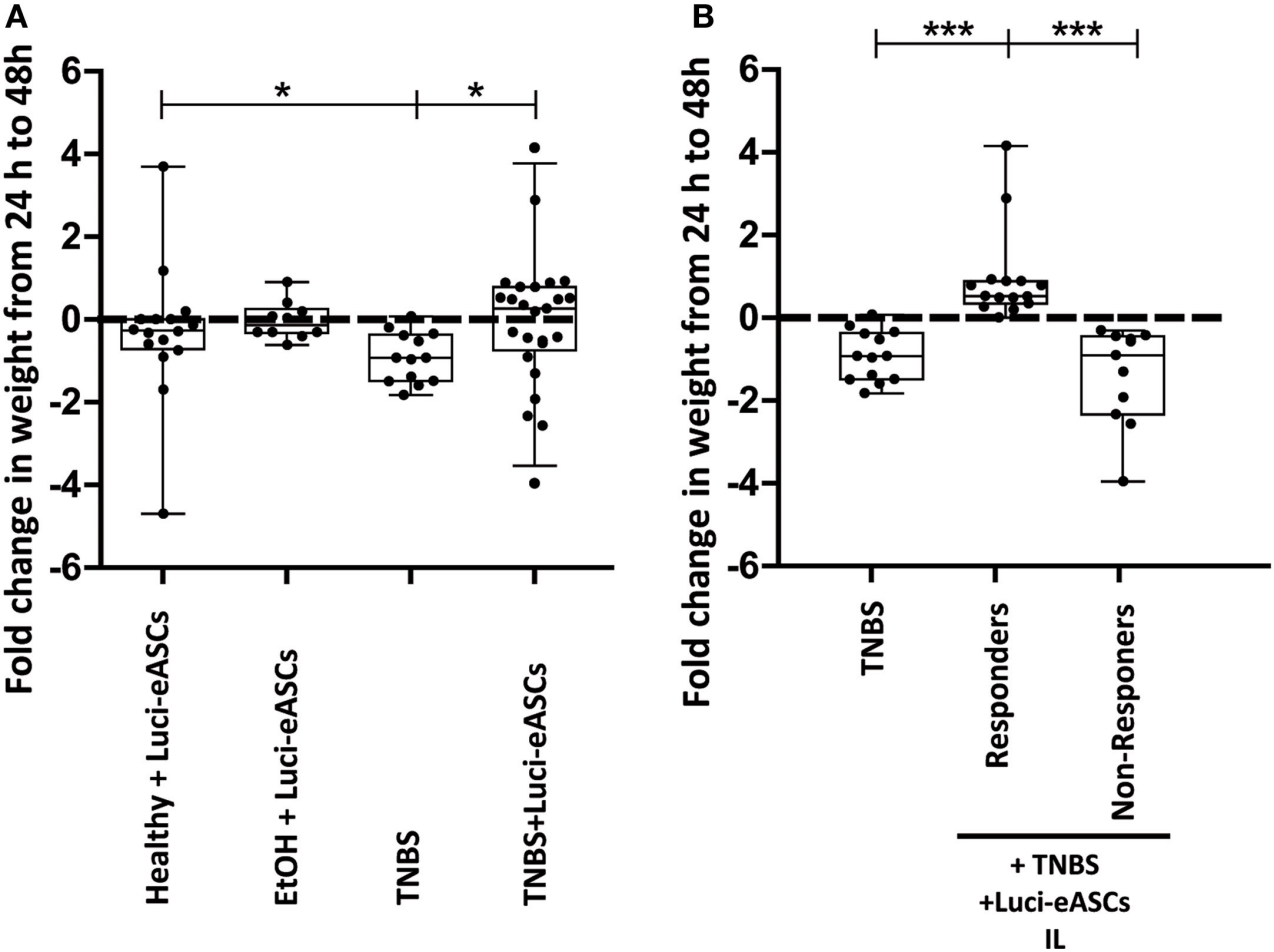
Body weight changes in healthy and TNBS-colitic mice. Fold change in body weight from 24 to 48 h after intranodal (IN) administration are represented. **(A)** As controls, mice infused with the Luci-eASC IN with or without intrarectal 50% EtOH (vehicle of the TNBS). Healthy + Luci-eASCs IN, *n* = 16; EtOH + Luci-eASCs, *n* = 10; TNBS, *n* = 14; TNBS + Luci-eASCs IN = 26. **(B)** Stratification of *in vivo* responses to treatment with Luci-eASCs administration IN in TNBS-colitic mice according to the fold change in body weights from 24 to 48 h. Colitic mice treated with Luci-eASCs were grouped into “responders” (Rs mice that did not lose weight at 48 h in comparison with 24 h) and “non-responders” (NRs mice that lost weight at 48 h in comparison with 24 h). TNBS, *n* = 14; TNBS + Luci-eASCs IN Rs, *n* = 15 and TNBS + Luci-eASCs IN NRs, *n* = 11. Data are presented by dots and box-plots that represent the interquartile range (p75, upper edge; p25, lower edge; p50, midline; p95, line above the box; and p5, line below the box) of the weight fold change at 48 h in comparison with the day 0. Significance was analyzed by the Mann–Whitney *U* test and represented by *p < 0.05 and ***p < 0.001. Results correspond to four independent experiments.

To investigate if the IN administration of Luci-eASCs can modulate acute inflammation, colitis was induced by administration of TNBS. One hour after the inflammatory challenge was induced, Luci-eASCs were infused IN. Body weights of mice were monitored within the first 48 h as the most meaningful physiological parameter for monitoring acute intestinal inflammation. As shown in Figure 3A, TNBS-treated mice had a significant reduction in their body weights [−0.92 (−1.49 to −0.36)] fold weight change in comparison with control mice [healthy mice, −0.26 (−0.71 to 0.00) fold body weight change and mice treated with intrarectal 50% EtOH as the vehicle for the TNBS, −0.13 (−0.32 to 0.25) fold body weight change]. The group of colitic mice treated with Luci-eASCs did not lose weight at 48 h after the inflammatory challenge [0.26 (−0.74 to 0.79)] in comparison to the untreated colitic mice.

These results demonstrate that, for the first time, the IN administration of eASCs is feasible and that can modulate efficiently acute intestinal inflammatory responses.

### Increased Amount of Luci-eASCs Within the Lymphatic System Can Be Correlated with an Improved Immunomodulation

To investigate whether a correlation between the biodistribution of the Luci-eASCs and the modulation of the inflammation exists, we used body weight changes between 24 and 48 h postinfusion of the Luci-eASCs as a parameter to stratify mouse responses to treatment with Luci-eASCs. In this sense, “responder” mice were those TNBS-colitic mice that did not lose, or even gained, weight between 24 and 48 h postinfusion of the Luci-eASCs [0.53 (0.34–0.89) in comparison with the TNBS-colitic mice, −0.92 (−1.49 to −0.36)]. “Non-responder” mice were those TNBS-colitic mice that continued to lose weight 48 h after the infusion of the Luci-eASCs [−0.91 (−2.34 to −0.45)] in comparison with the colitic mice. According to this stratification criteria, when Luci-eASCs were administered IN, 15 out of the 26 mice did not lose weight (responder mice). This means that 58% of mice treated with Luci-eASCs had a positive response to the treatment with Luci-eASCs (Figure 3B).

**Figure 4:**
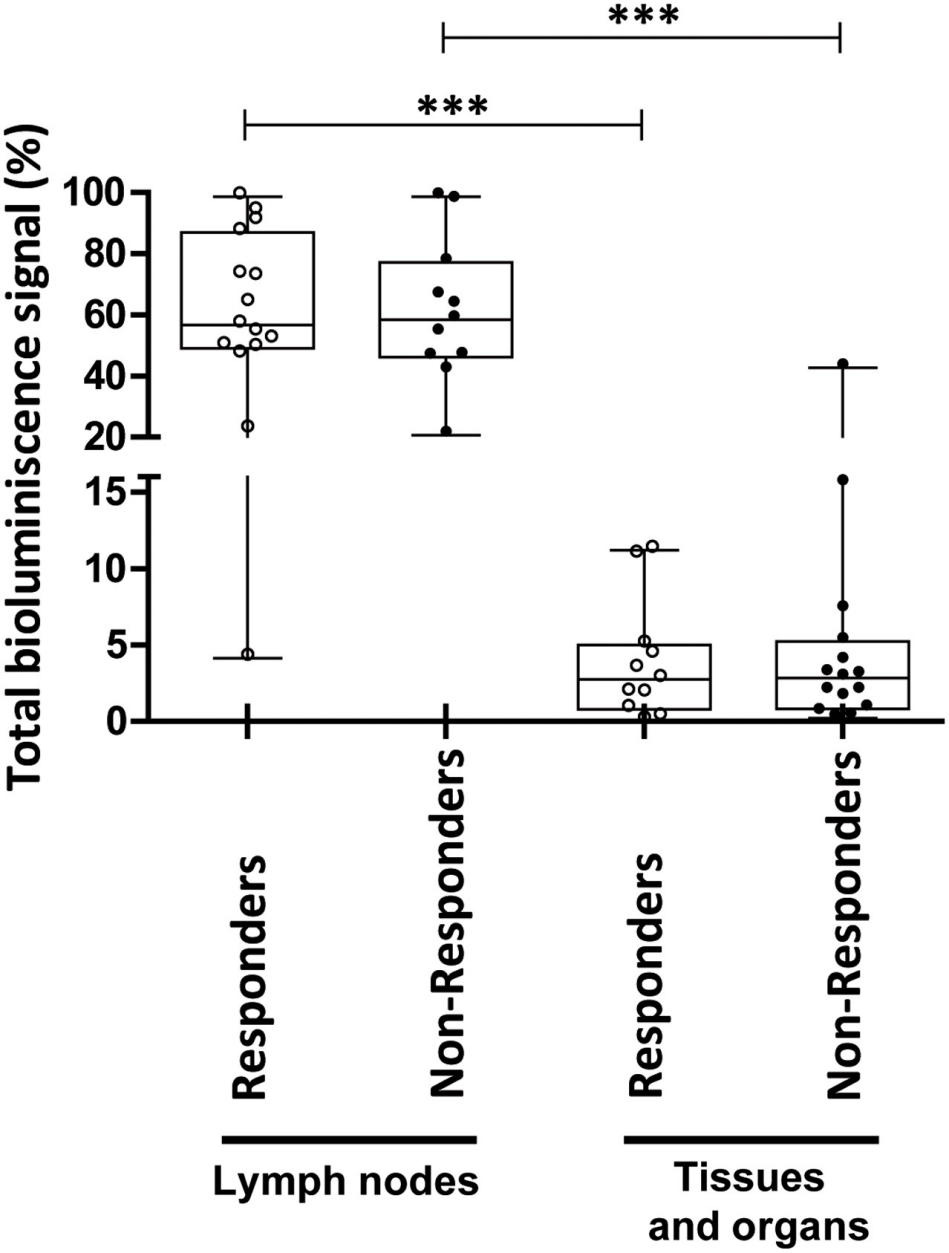
Analysis of total bioluminescence signal of Luci-eASCs in lymph nodes (LNs) and in tissues and organs at 48 h in “responder” and “non-responder” TNBS-colitic mice treated with Luci-eASCs. Sum of bioluminescence signals measured at 48 h as percentage of light units per LN (inguinal, popliteal, parathymic, parathyroid, mesenteric, caudal, and axillary included) and per each tissue and organ (liver, spleen, intestine, lungs, heart and blood included) relative to the total number of light units per mouse were analyzed. Healthy + Luci-eASCs intranodal (IN), *n* = 13; TNBS + Luci-eASCs IN Rs *n* = 15; TNBS + Luci-eASCs IN NRs *n* = 11. Data are presented by dots and box-plots that represent the interquartile range (p75, upper edge; p25, lower edge; p50, midline; p95, line above the box; and p5, line below the box) of the percentage of total bioluminescence signal. Significance was analyzed by the Mann–Whitney *U* test and represented by ***p < 0.001. Results correspond to four independent experiments.

Additionally, we compared the bioluminescence signals in the tissues and organs and in the LNs in the “responder” and “non-responder” mice. As shown in Figure 4, no differences in the total bioluminescence signal were found neither in the LNs [56 (49.1–87.0)% in responder mice and 58.4 (46.1–77.1)% in non-responder mice] nor in the other tissues and organs studied [2.8 (0.8–5.2)% in responder mice and 2.8 (0.8–5.0)% in non-responder mice], suggesting the lack of correlation between the modulation of acute inflammation in the intestine and the bioluminescence signal in LNs and other tissues and organs.

**Figure 5:**
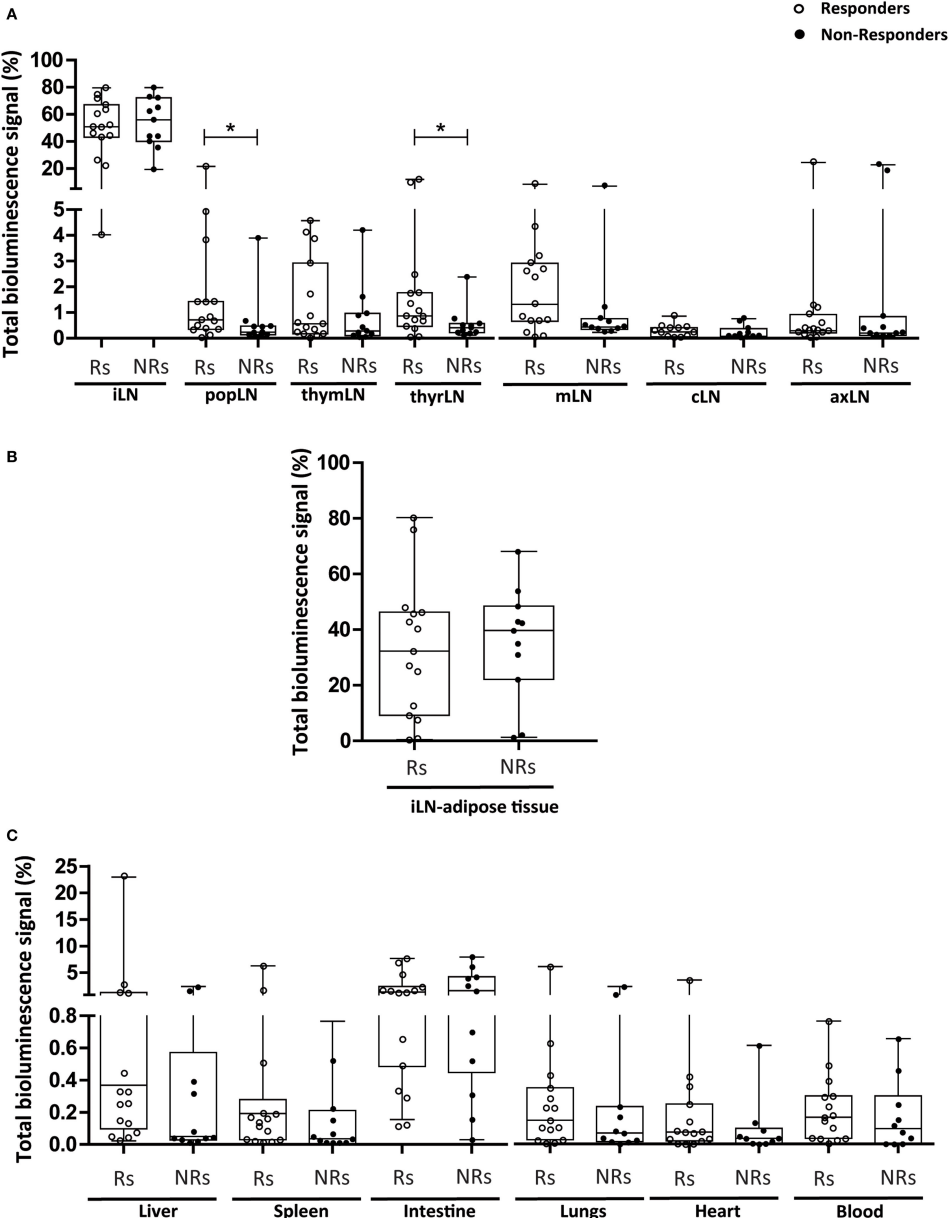
Analysis of total bioluminescence signal of Luci-eASCs in lymph nodes (LNs) (inguinal, popliteal, parathymic, parathyroid, mesenteric, caudal, and axillary), adipose tissue adjacent the inguinal LNs and in tissues and organs (the liver, spleen, intestine, lungs, heart, and blood) in “responder” and “non-responder” TNBS-colitic mice treated with Luci-eASCs at 48 h. Bioluminescence signals, measured at 48 h in the inguinal LNs (iLN), popliteal (popLN), parathymic (thymLN), parathyroid (thyrLN), mesenteric (mLN), caudal (cLN), and axillary (axLN) **(A)**, the adipose tissue surrounding the iLNs **(B)**, and in the liver, spleen, intestine, lungs, heart, and blood **(C)** in “responders” mice (that did not lose weight from 24 to 48 h) and “non-responders” mice (that lost weight from 24 to 48 h) were analyzed as percentage of light units per tissue relative to the total number of light units per mouse. Healthy + Luci-eASCs intranodal (IN), *n* = 12; TNBS + Luci-eASCs IN Rs *n* = 15; TNBS + Luci-eASCs IN NRs *n* = 11. Data are presented by dots and box-plots that represent the interquartile range (p75, upper edge; p25, lower edge; p50, midline; p95, line above the box; and p5, line below the box) of the percentage of total bioluminescence signal. Significance was analyzed by the Mann–Whitney *U* test and represented by *p < 0.05. Results correspond to four independent experiments.

When the bioluminescence signal of Luci-eASCs was analyzed at the injection site, no significant differences were found between the responder and the non-responder mice, neither in the iLNs [50.8 (43.1–67.0)% for responders; 56.0 (40.0–72.3)% for non-responder mice] nor in the iLN-adipose tissue [32.3 (9.2–47.0)% for the responder mice, 40.0 (22.1–48.5)% for the non-responder mice, Figures 5A,B].

In contrast to this, the bioluminescence signals were tended to increase in the popliteal and parathyroid LNs of the responder mice in comparison with the non-responder mice [0.7 (0.3–1.4) vs. 0.2 (0.1–0.5)% in popliteal and 0.9 (0.5–1.8) vs. 0.4 (0.2–0.6)% in the thyroid LNs, Figure 5A]. Also, the bioluminescence signal increased, but not significantly, in the parathymic and mesenteric LNs of the responder mice in comparison with the non-responder mice [0.56 (0.17–2.92) vs. 0.28 (0.10–0.97)% in parathymic and 1.32 (0.66–2.920) vs. 0.44 (0.34–0.76)% in the mesenteric LNs, Figure 5A]. This suggests that, in some instances, the increase amount of Luci-eASCs within the lymphatic system can be correlated with a better immunomodulation of the inflammatory responses.

However, no clear differences were found in the bioluminescence signals in tissues and organs analyzed between responder and non-responder mice. Although, a tendency to increase the bioluminescence signal was found in the liver, spleen, lungs, heart and blood of responder mice in comparison with the non-responder mice. Also, there was a significant increase in the bioluminescence signal in the liver [0.37 (0.10–1.26)%] in comparison with the heart [0.17 (0.03–0.30)%] of the responder mice but not in the non-responder mice [0.05 (0.03–0.57) vs. 0.090 (0.002–0.299)%, Figure 5C].

Overall, these data indicate that, in responder mice, an increase amount of Luci-eASCs is located within the lymphatic system as suggested by the increase bioluminescence signals found in the popliteal, parathymic, parathyroid, and mesenteric LNs in comparison with the non-responder mice. However, in the tissues and organs analyzed these differences were not observed, although increased bioluminescence signals were found in the inflamed intestine.

## Discussion

The functional properties of the MSCs have become of great interest for the treatment of a variety of diseases (20, 47, 48). At present, it is unknown whether the therapeutic effects of MSCs are carried out by a small fraction of the administered MSCs that migrates to sites of inflammation or/and through distant induction of regulatory immune cells that, rapidly and subsequently, mediate immunomodulation and tissue repair (22). In any case, their therapeutic effects are linked to a final increase of myeloid and lymphoid cells with a regulatory phenotype in the secondary lymphoid tissues associated to the inflammation site (18, 19, 25, 26). Thus, we proposed, for the first time, the administration of eASCs directly into the LNs, in order to determine whether the IN administration of MSCs could be an alternative strategy to modulate inflammation. The IN (or intralymphatic) route is being extensively used in immunotherapy protocols for treatment of cancer (29–32) and allergy (33, 34). The data reported indicate that this route of administration is feasible and can be translated to the clinic (35–37). Additionally, Gil-Ortega et al. provided robust evidences that endogenous adipose stromal cells can traffic *in vivo* through lymphatic system and home to the LNs (28).

In this study, we observed that IN administration of Luci-eASCs can modulate acute intestinal inflammation in a mouse model of colitis induced by TNBS as shown by the maintenance of the body weights of colitic mice at 48 h after infusion of the cells (Figure 3). To our knowledge, this is the first time that a single dose of 3.2 × 10^5^ eASCs show immunomodulatory effects in a mouse model of colitis induced by TNBS (10, 40, 49–52). We cannot provide any reasonable explanation for the observation that 58% of eASC-treated mice have a positive response to the cell therapy. It should be noticed that the biodistribution analysis was conducted using a single IL infusion of the Luci-eASCs whereas the majority of studies aiming at modulating immune responses used multiple eASCs doses to achieve robust modulation of inflammation. Since the main goal of our study was to define the *in vivo* biodistribution of eASCs infused intranodally in an inflammatory environment, we cannot exclude that multiple IL infusion of eASCs would achieve a complete response.

Furthermore, Mancheño-Corvo et al. demonstrated that eASCs administered IN reduced systemic inflammation, in an experimental model of arthritis, by inducting CD25^+^Foxp3^+^CD4^+^ cells and IL10^+^CD4^+^ cells in the LNs and in the spleen (27).

*In vivo* biodistribution of MSCs is an important parameter to take into account when evaluating the efficacy of the cell therapy with MSCs. After intravenous administration, the most widely used route for MSCs in the clinic, the infused MSCs accumulate preferential in the lungs (24, 38) and this is generally accompanied by a transient immunomodulatory effect (22, 26, 53–56) due to their short-term persistence *in vivo* regardless of their origin (syngeneic, allogeneic or, even, xenogeneic). This, obviously, might impact the bioavailability of the cells, their targeting to tissues and, probably, their long-term therapeutic efficacy (24). Thus, in this study, we proposed to study the biodistribution of the IN administration of MSCs which potentially may represent an alternative route of administration for MSCs. The number of MSCs that can be delivered inside the LN after IN administration is significantly greater than the number of cells that reach the LNs following their delivery through the blood stream. This may allow achieving therapeutic effects using a low dose of MSCs. This will have major implications to improve the safety of the cell therapy together with a significant reduction in the production costs.

In our study, we observed that, following the IN administration of the Luci-eASCs, the majority of the bioluminescence signal was found in the LNs (Figure 1) mainly at the injection site, either in the inguinal LNs or in the adipose tissue surrounding the iLNs where the cells were injected (Figures 2A,B; Figure S2C in Supplementary Material). Only 0.4 (0.2–1.3)% of the bioluminescence signal was found in other locations within the lymphatic system with no clear preference for any of the LNs analyzed (Figure 2A). These results show that, by the IN administration of MSCs, a higher number of cells are within the lymphatic system when compared to intravenous administration (25, 41) which could improve their therapeutic effects.

Only 1.9 (0.4–4.1)% of the bioluminescence signal was found in the tissues and organs analyzed and, surprisingly, in TNBS-colitic mice, a significant increase of the total bioluminescence signal was found with in comparison with the healthy mice. This paralleled with a decrease in the bioluminescence signal in the iLNs and in the adipose tissue surrounding the iLNs of TNBS-colitic mice when compared to healthy mice (Figures 1 and 2), suggesting a rapid and preferential trafficking of the Luci-eASCs to the inflamed tissues and organs upon their IN administration. This increase of the bioluminescence signal corresponded mainly to the intestine and to a lesser extent to the liver, spleen, lungs, and blood of the Luci-eASC-treated colitic mice suggesting that, by the IN route, MSCs have a preferential migration to the inflamed tissues, similar to what was happened when MSCs are infused by other routes of administration (23–25).

Trying to know if the presence of the MSCs at the inflammation site is necessary for the modulation of the immune responses, we investigated the correlation between the biodistribution of the Luci-eASCs and the modulation of the inflammation attending to the stratification criteria into “responders” and “non-responders” mice. These stratification criteria were done based on the body weights of the Luci-eASC-treated colitic mice. The increase amount of Luci-eASCs within the lymphatic system in the responder mice can be correlated with an improved immunomodulation (Figure 5). This increase amount of Luci-eASCs within the lymphatic system was preferentially found in the popLNs, thyrLNs, thymLNs, and mLNs. We did not see any correlation between the presence of the MSCs in the tissues and organs analyzed and the modulation of the inflammation, this result suggests that the presence of the Luci-eASCs in the intestine does not seem to be necessary to achieve an adequate modulation of the inflamed intestine.

In summary, all these results suggest that the presence of the Luci-eASCs within the lymphatic system is responsible for their immunomodulatory effects. The IN administration of the Luci-eASCs allows the trafficking of a small proportion of cells to the inflamed intestine; although, a direct correlation with their biological effects could not be established. Further, these results confirm our hypothesis that the immunomodulatory effects of the eASCs may take place in the LNs where the immunoresponses are orchestrated.

Based on these results and those reported in the accompanying manuscript (27), further investigations are warranted to assess the beneficial effect of the IN route in cell therapy protocols with eASCs for treatment of immunomediated disorders.

## Conclusion

These data demonstrate the feasibility of using the IN route of administration to infuse eASCs for the treatment of intestinal inflammatory diseases since Luci-eASCs can modulate an acute intestinal inflammation.

After IN administration, the majority of the Luci-eASCs remained in the inguinal LNs where the cells were infused and only lower levels of bioluminescence signals were found in tissues and organs related to the inflammation. After intestinal inflammation, an increased trafficking of the Luci-eASCs to the inflamed organs was found. In some instances, the increase amount of Luci-eASCs within the lymphatic system can be correlated with an improved immunomodulation. Taken together, these results indicate that the IN administration of eASCs represents a novel route for administering eASCs in cell therapy protocols.

## Ethics Statement

Institutional Animal Care and Use Committee at University of Albacete (Spain) and Spanish Ethics Committee of reference (Clinica de la Luz Hospital) for the site of tissue procurement.

## Author Contributions

Conception and/or design of the work: ML-S, OD, JA, DB, JR, JB, WD, EL, and MG. Acquisition of data for the work: ML-S, PM-C, AE, RM, JA, EL, and MG. Analysis and interpretation of data for the work: ML-S, PM-C, AE, RM, OD, JA, DB, JR, JB, WD, EL, and MG. Manuscript writing: ML-S, PM-C, and MG. Revised manuscript for important intellectual content: AE, RM, OD, DB, JR, JB, and WD. All authors reviewed the manuscript and gave final approval for the work.

## Conflict of Interest Statement

PM-C, RM, OD, WD, and EL are full-time employees of TiGenix, DB is full-time employee of Grifols, and JA is full-time employee of Coretherapix.

## Supplementary Material

Figure S1 Characterization of Luci-eASCs. **(A)** Luci-eASCs were characterized by flow cytometry using antibodies against CD73, CD90, CD105, CD14, CD34, CD45, and HLA-DR (gray color). The corresponding isotype controls for each surface marker are shown (white color). Histograms shown are representative of three experiments. **(B)** Representative histograms of T-lymphocyte proliferation are shown. CFSE-labeled peripheral blood mononuclear cells (PBMCs) were stimulated with anti-CD3/CD2/CD28-coated beads in the absence (gray color) or presence (white color) of transduced ASCs (Luci-eASCs, left) or untransduced eASCs (right), ratio 1:25 eASCs or Luci-eASC:PBMCs. Proliferation of the viable CD3^+^ T cells was monitored after 120 h by flow cytometry.

Figure S2 *In vivo* imaging of Luci-eASCs after IN infusion of Luci-eASCs. **(A)** Representative images of total bioluminescence signal at 24 and 48 h when Luci-eASCs were administered intranodally in healthy mice. **(B)** Graph represents the decline of total bioluminescence signal (luciferase activity) at different time points after 3.2 × 10^5^ Luci-eASCs were administered intranodally into healthy mice. **(C)** Representative images of bioluminescence signal at 48 h of left inguinal (iLN), right inguinal lymph nodes (LNs), popliteal (popLN), parathyroid (thyrLN), parathymic (thymLN), mesenteric (mLN), and axillary (axLN) lymph nodes. **(D)** Representative images of bioluminescence signal at 48 h in intestine, blood, lungs, heart, liver, spleen, and adipose tissue adjacent inguinal lymph nodes (iLNs AT).
