# Supplementary figures and images for: Biodistribution and Efficacy of Human Adipose-Derived Mesenchymal Stem Cells Following Intranodal Administration in Experimental Colitis
Source: Front Immunol. 2017 Jun 8;8:638. doi: 10.3389/fimmu.2017.00638 (PMC5462906; doi:10.3389/fimmu.2017.00638)

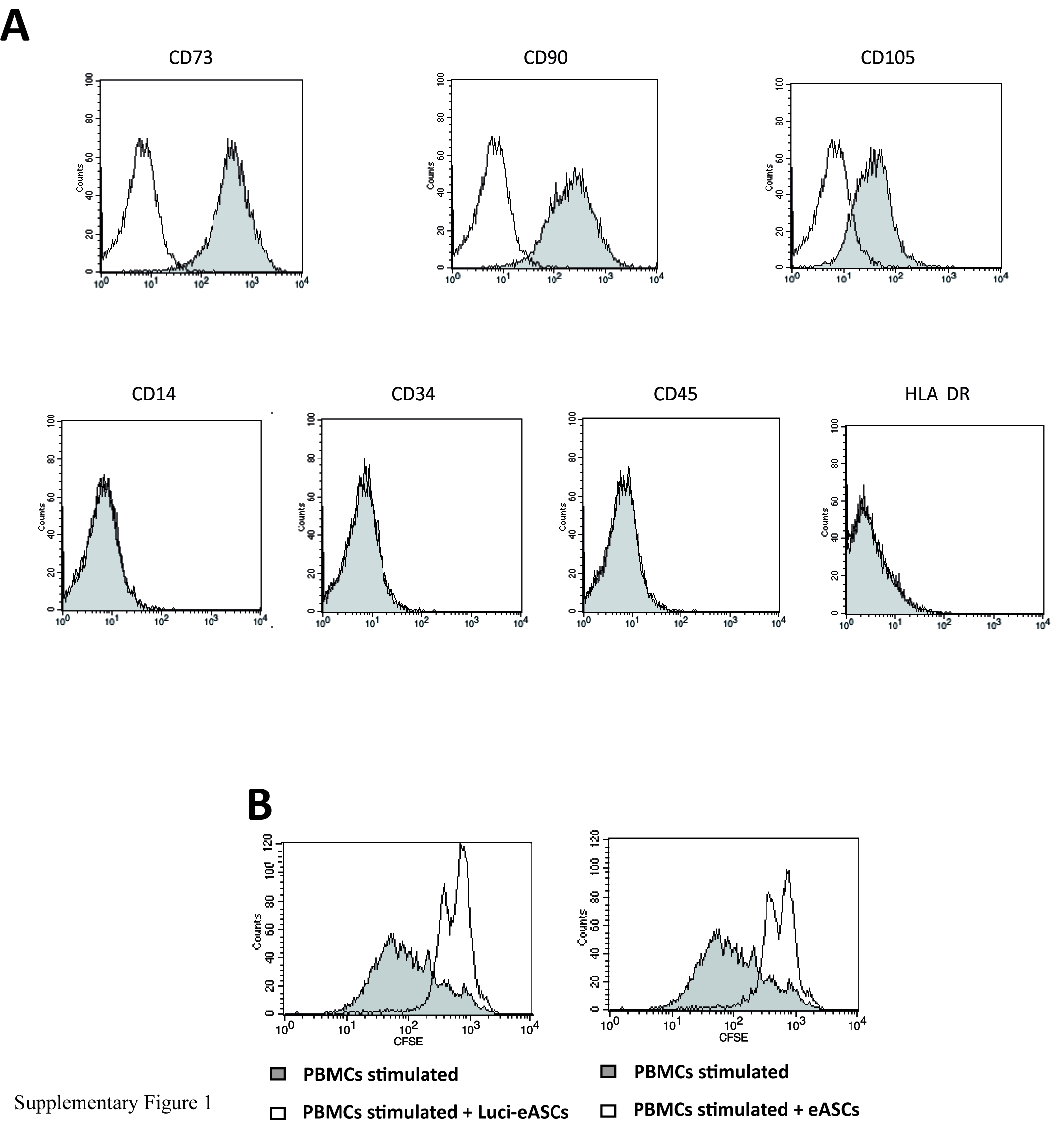

Supplement: Figure S1 — Characterization of Luci-eASCs. (A) Luci-eASCs were characterized by flow cytometry using antibodies against CD73, CD90, CD105, CD14, CD34, CD45, and HLA-DR (gray color). The corresponding isotype controls for each surface marker are shown (white color). Histograms shown are representative of three experiments. (B) Representative histograms of T-lymphocyte proliferation are shown. CFSE-labeled peripheral blood mononuclear cells (PBMCs) were stimulated with anti-CD3/CD2/CD28-coated beads in the absence (gray color) or presence (white color) of transduced ASCs (Luci-eASCs, left) or untransduced eASCs (right), ratio 1:25 eASCs or Luci-eASC:PBMCs. Proliferation of the viable CD3+ T cells was monitored after 120 h by flow cytometry. [file Image_1.TIF]

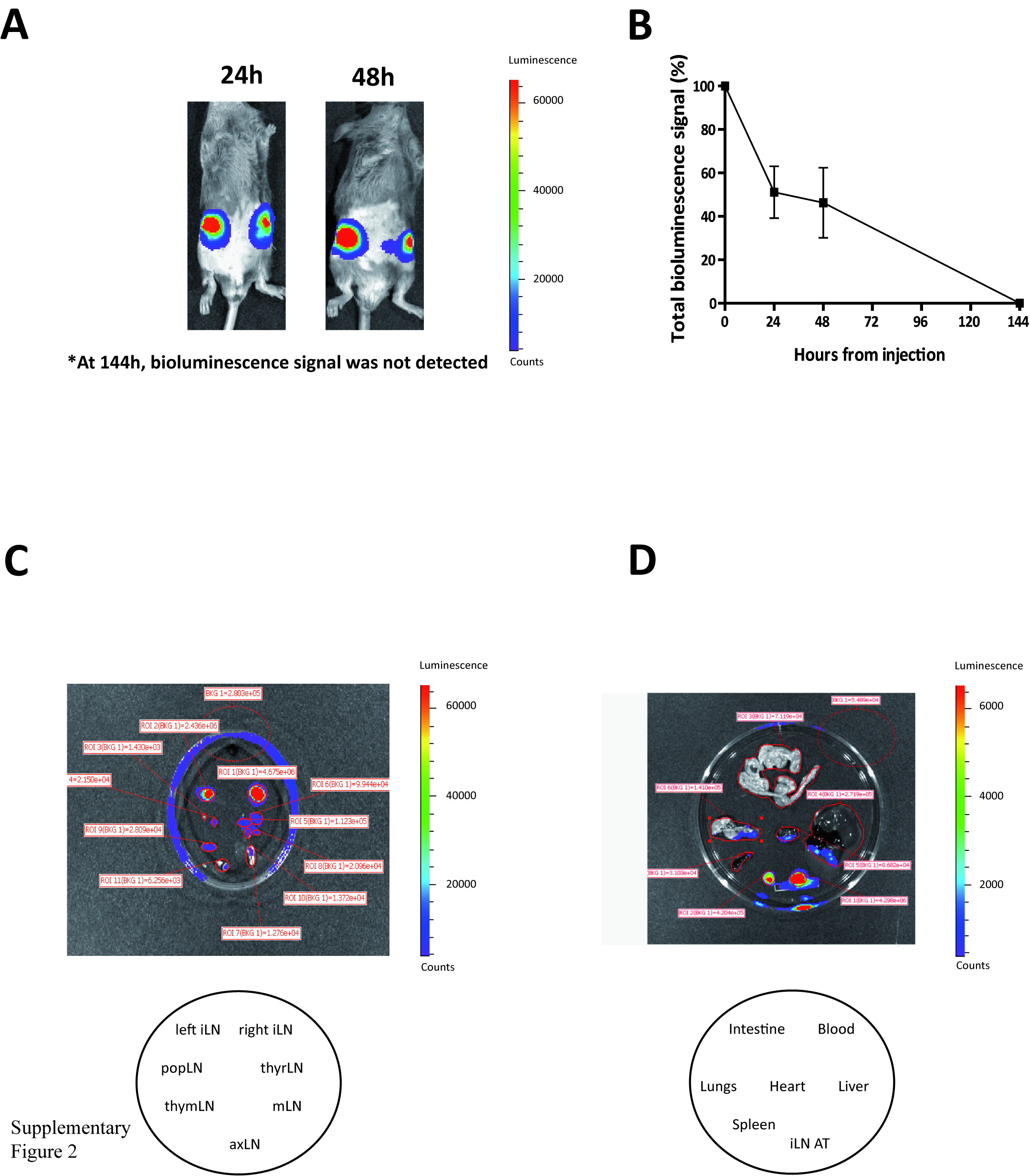

Supplement: Figure S2 — In vivo imaging of Luci-eASCs after IN infusion of Luci-eASCs. (A) Representative images of total bioluminescence signal at 24 and 48 h when Luci-eASCs were administered intranodally in healthy mice. (B) Graph represents the decline of total bioluminescence signal (luciferase activity) at different time points after 3.2 × 10^5^ Luci-eASCs were administered intranodally into healthy mice. (C) Representative images of bioluminescence signal at 48 h of left inguinal (iLN), right inguinal lymph nodes (LNs), popliteal (popLN), parathyroid (thyrLN), parathymic (thymLN), mesenteric (mLN), and axillary (axLN) lymph nodes. (D) Representative images of bioluminescence signal at 48 h in intestine, blood, lungs, heart, liver, spleen, and adipose tissue adjacent inguinal lymph nodes (iLNs AT). [file Image_2.TIF]
